# Green Synthesis of Metal and Metal Oxide Nanoparticles and Their Effect on the Unicellular Alga *Chlamydomonas reinhardtii*

**DOI:** 10.1186/s11671-018-2575-5

**Published:** 2018-05-23

**Authors:** Nhung H. A. Nguyen, Vinod Vellora Thekkae Padil, Vera I. Slaveykova, Miroslav Černík, Alena Ševců

**Affiliations:** 1 Institute for Nanomaterials, Advanced Technologies and Innovation, Technical University of Liberec, Studentská 2, 461 17 Liberec, Czech Republic; 2 Faculty of Sciences, Earth and Environmental Sciences, Institute F.-A. Forel, University of Geneva, Uni Carl Vogt, 66 Bvd Carl-Vogt, 1211 Geneva, Switzerland

**Keywords:** Green chemistry, Metal nanoparticles, Biological effect, *Chlamydomonas reinhardtii*

## Abstract

Recently, the green synthesis of metal nanoparticles has attracted wide attention due to its feasibility and very low environmental impact. This approach was applied in this study to synthesise nanoscale gold (Au), platinum (Pt), palladium (Pd), silver (Ag) and copper oxide (CuO) materials in simple aqueous media using the natural polymer gum karaya as a reducing and stabilising agent. The nanoparticles’ (NPs) zeta-potential, stability and size were characterised by Zetasizer Nano, UV–Vis spectroscopy and by electron microscopy. Moreover, the biological effect of the NPs (concentration range 1.0–20.0 mg/L) on a unicellular green alga (*Chlamydomonas reinhardtii*) was investigated by assessing algal growth, membrane integrity, oxidative stress, chlorophyll (*Chl*) fluorescence and photosystem II photosynthetic efficiency. The resulting NPs had a mean size of 42 (Au), 12 (Pt), 1.5 (Pd), 5 (Ag) and 180 (CuO) nm and showed high stability over 6 months. At concentrations of 5 mg/L, Au and Pt NPs only slightly reduced algal growth, while Pd, Ag and CuO NPs completely inhibited growth. Ag, Pd and CuO NPs showed strong biocidal properties and can be used for algae prevention in swimming pools (CuO) or in other antimicrobial applications (Pd, Ag), whereas Au and Pt lack these properties and can be ranked as harmless to green alga.

**Electronic supplementary material:**

The online version of this article (10.1186/s11671-018-2575-5) contains supplementary material, which is available to authorized users.

## Background

Metal and metal oxide nanoparticles (NPs) have received substantial research attention due to their exceptional electrical, optical, magnetic and catalytic properties. These have enabled their broad use in diverse industrial, medical, agricultural and environmental applications, with further uses constantly under development [1–4]. Traditional synthesis methods for pristine metal and metal oxide NPs include reducing and stabilising chemical agents that are toxic to humans and to other species in different trophic levels [5–11]. In response, researchers are now looking for alternative “green synthesis” approaches in an effort to reduce or eliminate harmful chemicals during the production of NPs [12–18].

Many studies have reported on the wide range of metal and metal oxide NP applications, owing to their unique and wide-ranging physicochemical properties [19]. Silver (Ag) NPs, for example, are widely used for medical, textile, food packaging and water treatment applications [20–23]. Gold (Au) NPs have been employed in biomedical research, while platinum (Pt) NPs are widely used for industrial applications due to their catalytic properties [24, 25]. Finally, palladium (Pd) NPs have been used as catalysts during the manufacture of pharmaceuticals [26, 27] and copper oxide (CuO) NPs as antifouling agents in paints and fabrics due to their proven antibacterial properties [28]. Metal NPs can serve as catalysts for degrading a wide variety of common environmental contaminants, including polychlorinated biphenyls (PCBs), halogenated aliphatics, organochlorine pesticides, toxic metals and halogenated organic solvents [29]. CuO, Ag and Au NPs are also used for sensing poisonous gases, such as carbon monoxide (CO), hydrogen cyanide (HCN) and sulphur dioxide (SO_2_) [30, 31]. Recently, a number of metal NPs (Au, Ag and Cu) that exhibit localised surface plasmon resonance have been used in the development of bio-nanosensors [24].

Unfortunately, metal and metal oxide NPs have the potential to negatively impact both human health and the environment in general, e.g. by generating new classes of toxins that can adversely affect microbial communities, with knock-on effects for the whole ecosystem [32–35]. As a result, the effects of NPs on microorganisms have been widely studied. Ag NPs, for example, have been shown to inhibit algal growth and photosynthesis, changing the chlorophyll (*Chl*) fluorescence content of *Chlamydomonas reinhardtii* [36], altering the cellular growth of *Thalassiosira pseudonana* and *Synechococcus* sp. [37] and affecting growth and cellular viability in the aquatic plant swollen duckweed *Lemna gibba* [38]**.** Książyk et al. [39] and Sørensen et al. [40] have reported Pt NPs as inhibiting growth in the freshwater microalgae *Pseudokirchneriella subcapitata* [39, 40]. Unsurprisingly, both Ag and Pd NPs have been applied as useful antibacterial agents against a variety of Gram-positive and Gram-negative bacteria [41–43]. In contrast, Au NPs are thought not to have a negative impact on bacteria or algae [44, 45], though one study has shown that they can be toxic, depending on their charge and surface chemistry [46]. Negative impacts have been reported for CuO NPs on *C. reinhardtii* [36, 47], *P. subcapitata* [48], western waterweed *Elodea nuttallii* [49] duckweed *Lemna* sp*.*, *Daphnia magna* [48] and the early life stages of zebrafish *Danio rerio* [50, 51].

Metal NPs possess physical and chemical properties that can cause cell damage, e.g. through excessive generation of reactive oxygen species (ROS) with subsequent damage to DNA, proteins and lipids. The formation of ROS by Ag NPs has been detected in *Chlorella vulgaris* and *Dunaliella tertiolecta* cultures and in *L. gibba* [52], as well as in bacteria [53]. CuO and Fe NPs are both able to generate hydrogen radicals, a family of ROS produced via the Fenton reaction, which can harm a variety of aquatic and terrestrial organisms [54, 55].

Green chemistry is a set of principles or practices that encourage the design of products and processes that reduce or eliminate the use and generation of hazardous substances [56–58]. Current green nanotechnology practices often involve the use of natural sources, non-hazardous solvents, biodegradable and biocompatible materials and energy-efficient processes in the preparation of NPs [59]. As an example, biopolymers, such as cellulose, chitosan, dextran or tree gums, are often used as reducing and stabilising agents for metal NP synthesis [12, 60–62]. Gum karaya (GK) used in this study is a natural tree gum from *Sterculia* consisting of approximately 13–26% galactose and 15–30% rhamnose, 30–43% galacturonic acid, 37% uronic acid residues and about 8% acetyl groups [63]. Toxicological studies proved GK as non-toxic, allowing its use even as a food additive [62–65].

In this study, we aimed to use a green chemistry approach to prepare a number of metal (Ag, Au, Pt, Pd) and metal oxide (CuO) NPs using aqueous solutions of a natural polymer, GK. The biological effect of these newly prepared NPs was investigated on *C. reinhardtii* using a range of cellular responses, including algal growth, oxidative stress, membrane damage, *Chl* fluorescence and photosynthesis. NP stability, size and zeta potential were determined in algal growth medium, along with solubility and abiotic testing of ROS generation.

## Methods

### Materials

Commercial GK, silver nitrate (AgNO_3_), hydrogen tetrachloroaurate (HAuCl_4_·3H_2_O), copper chloride (CuCl_2_·2H_2_O), chloroplatinic acid (H_2_PtCl_6_), potassium tetrachloropalladate(II) (K_2_PdCl_4_), hydrogen chloride (HCl), sodium hydroxide (NaOH) and ammonium hydroxide (NH_4_OH) were all purchased from Sigma-Aldrich, USA. Deionised (DI) water was used for all experiments. All chemicals and reagents used in this study were analytical grade.

The *C. reinhardtii* algal culture (strain CPCC11) was obtained from the Canadian Phycological Culture Centre (CPCC, Department of Biology, University of Waterloo, Canada).

### GK Processing

GK powder (1 g) was introduced into a glass beaker containing 1 L of DI water and gently stirred overnight on a magnetic stirrer. The gum solution was subsequently left at room temperature (20 °C) for 18 h to separate out any undissolved matter. The gum solution was then filtered through a sintered glass funnel (10–16 μm pore size) and the clear solution lyophilised and stored until needed.

### Synthesis of Metal and Metal Oxide NPs Using GK

Briefly, 100 μL aliquots of 10 mM AgNO_3_, HAuCl_4_, H_2_PtCl_6_ and K_2_PdCl_4_ solutions were added to 10 mL of aqueous GK solution in separate 50-mL conical flasks. The pH of the colloidal dispersion was adjusted by adding 0.1 N HCl or 0.1 N NaOH in order to achieve maximum yield of NP formation. To synthesise the Ag, Au, Pt and Pd NPs, the AgNO_3_, HAuCl_4_, H_2_PtCl_6_, and K_2_PdCl_4_ and GK mixtures were agitated in an Innova 43 orbital shaker (New Brunswick Scientific, USA) at 250 rpm at temperatures ranging from 45 to 95 °C for 1 h. The solutions turned light yellow, wine red, intense black and muted black, respectively, indicating the formation of Ag, Au, Pt and Pd NPs. In the case of Pt, reduction and NP formation occurred at a pH of 8.0 and a temperature of 90 °C, while Pd NPs were formed at pH 8.5 and 95 °C. See more in Padil et al. [66, 67].

CuO NPs were synthesised using a colloid thermal synthesis process [13]. Briefly, 100 μL aliquots of a 10 mM solution of dihydrate copper chloride (CuCl_2_·2H_2_O) was mixed with 10 mL of the GK solution (100 mg dispersed in 10 mL of DI water) and NaOH in separate 50-mL conical flasks, with CuCl_2_·2H_2_O and NaOH maintained at a molar ratio of 2:5. The mixture containing the CuCl_2_·2H_2_O and GK was agitated at 250 rpm at a temperature of 75 °C for 1 h in an orbital shaker. The colour of the mixture gradually changed from bluish to black, indicating the formation of CuO NPs. The resulting precipitate was obtained by centrifugation and washed first with ethanol then DI water.

### Characterisation of Green-Synthesised NPs

The metal concentration within the freshly synthesised NPs was measured using inductively coupled plasma mass spectrometry (ICP-MS, OPTIMA 2100 DV, Perkin Elmer).

The formation and stability of the metal NPs were assessed using a Cintra 202 UV–Vis spectrophotometer (GBC, Australia), NP stability being determined after 6 months.

Transmission electron microscopy (TEM) images of the Ag, Au, Pt, Pd and CuO NPs were obtained using a Tecnai F 12 microscope (FEI, Thermo Fisher Scientific, Oregon, USA) operating at an acceleration voltage of 15 kV. Samples were prepared for TEM analysis by dropping 10–20 μL of GK-inorganic NP dispersion onto a copper grid and drying at room temperature, after removing excess solution.

### Algal Culture Conditions

*Chlamydomonas reinhardtii* was cultured in TAPx4 medium (Additional file 1: Table S1, supporting information) at 20 °C in an incubator (Infors, Switzerland) equipped with a shaker continuously rotating at 100 rpm and an illumination regime of 114.2 μmol phot m^−2^ s^−1^. The algal cells were grown at an exponential rate in order to obtain approximately 10^6^ cells/mL.

### Characterisation of NPs in Algal Exposure Medium

NP size distribution in the *C. reinhardtii* TAPx4 medium was measured using the differential centrifugal sedimentation technique (DCS) on a DC24000UHR disc centrifuge (CPS Instruments Inc., USA). Measurements were made at a disc rotation speed of 24,000 rpm, and particle sedimentation was performed using an 8–24% (*w*/*w*) sucrose density gradient. Prior to each sample measurement, the instrument was calibrated using PVC nanosphere standards (470 nm). The NPs were also characterised by electrophoretic mobility, and the Smoluchowski approximation used to determine zeta-potential (ZP) on a Zetasizer ZS (Malvern Instruments Ltd., UK). Each measurement was performed over 10 runs with autocorrelation functions of 10 s, each result being obtained from triplicate measurements of the same sample.

The ultra-filtration method was used to determine the amount of ion metal in the algal media (Cheloni et al. [47]; Ma et al. [68]). Aliquots drawn at different time intervals (2 and 24 h) were centrifuged for 30 min at 7500 rpm to separate the particles and aggregates. The supernatant was then filtered through Amicon Ultracel 3K ultra-filtration filters with a 3-kDa molecular weight cut-off (Millipore, USA) to separate ions from the particles. NPs and aggregates with a diameter larger than 1.3 nm were retained on the filter, and the filtrate was analysed by ICP-MS for dissolved ions [68].

Abiotic ROS generation with increasing concentration of NPs in algal medium was determined using fluorescent dichlorodihydroflourescein diacetate (H_2_DCF-DA, Sigma–Aldrich, Switzerland), as described in earlier studies [47, 69].

### Effect of NPs on Algal Growth, Membrane Integrity and Generation of Oxidative Stress

The effect of the metal and metal oxide NPs on algal growth, membrane integrity and generation of oxidative stress was tested using flow cytometry (FCM; BD Accuri C6 Flow Cytometer, BD Biosciences, USA). The experiment was performed in transparent vials (PS, 50 mL, Semadeni, Switzerland) containing 5 mL of algal suspension and NPs at concentrations of 1, 5, 10 and 20 mg/L. Control samples without NPs were run in parallel. Algal cells were heated in boiling water (100 °C) for 15 min in order to provide a positive control for damaged cell membranes. Algal cells were also treated with cumin (Sigma-Aldrich, USA), an oxidative species agent, for 30 min in the dark as a positive control of oxidative stress (ROS). All untreated samples and samples treated with NPs were incubated under similar conditions to those adopted for maintaining cultures. Sub-samples were taken after 1, 3, 5 and 24 h to assess the effect of NPs on cellular membrane integrity and oxidative stress using FCM. A 250-μL aliquot of each sample was transferred to a Microtiter® 96-well flat-bottom plate. To assess cellular membrane integrity, propidium iodide (PI) fluorescent probes (P4170, Sigma-Aldrich, USA) were added to the sample at a final concentration of 7 μM. For oxidative stress detection, CellROX® Green Reagent (ROS) (C10444, Life Technologies, USA) was added to the samples as per the product instructions. In short, PI binds to DNA and attaches to RNA following intracellular penetration through impaired cell membranes, but it is excluded from the healthy cells. CellROX® Green Reagent is a probe for measuring oxidative stress in live cells. The cell-permeant dye is weakly fluorescent while in a reduced state but exhibits bright green photostable fluorescence upon oxidation by ROS and subsequent binding to DNA. Thus, its signal is primarily localised on the nucleus and mitochondria. The plates were incubated in the dark for 20 min (PI) and 30 min (ROS) before FCM measurement. The algal suspensions were then passed through the FCM with a blue 488-nm excitation laser. CellROX Green was measured in the FL1 channel 533/30 nm, PI red fluorescence in the FL2 channel 585/40 nm and the red autofluorescence of chlorophyll a (*Chla*) in FL3 channel > 670 nm. The experiment was carried out in duplicate and repeated.

FCM data were analysed using CFlow Plus software (BD Biosciences, USA). The samples were gated, based on forward scattering properties and red autofluorescence of *Chla*, to eliminate signals from NPs, debris and other contaminants. The number of cells, percentage of damaged cell membranes or oxidatively stressed cells, and autofluorescence data were retrieved based on autofluorescence of *Chla* (670 nm), PI-labelled cells (585 nm) and ROS Green (533 nm) (Additional file 1: Figure S1).

### Efficiency of Algal Photosystem II

Metal and metal oxide NPs suspensions were added to the same algal culture (approx. 10^6^ cells/mL) in 15-mL glass flasks in order to achieve final concentrations of 1, 5, 10 and 20 mg/L. Algal cultures without NPs were prepared as negative controls. All samples were then transferred to an incubator under the same conditions used for the original algal cultures. Aliquots (2.2 mL) of each sample were taken immediately and after 1, 3, 5 and 24 h incubation in order to detect the photosystem II quantum yield (QY) using an AquaPen-C AP-C 100 fluorometer (PSI Ltd., Czech Republic). All measurements were undertaken in triplicate. QY represents the ratio of variable fluorescence (*F*_v_ = *F*_m_ − *F*_0_) to maximum fluorescence (*F*_m_), with QY = *F*_v_:*F*_m_ used as a proxy of photochemical quenching efficiency [70]. *F*_m_ was obtained by applying illumination at 680 nm for a few seconds before and at the end of illumination, with minimal fluorescence (*F*_0_) being the initial measurement at the minimum fluorescence level in the absence of photosynthetic light.

### Statistical Analysis

The effect of metal and metal oxide NPs on *C. reinhardtii* were tested using analysis of variance ANOVA and Dunnett’s test (GraphPad PRISM, USA). Significance levels were set at *P < 0.05, **P < 0.01 and ***P < 0.001.

## Results

### Formation and Primary Characterisation of NPs

**Fig. 1:**
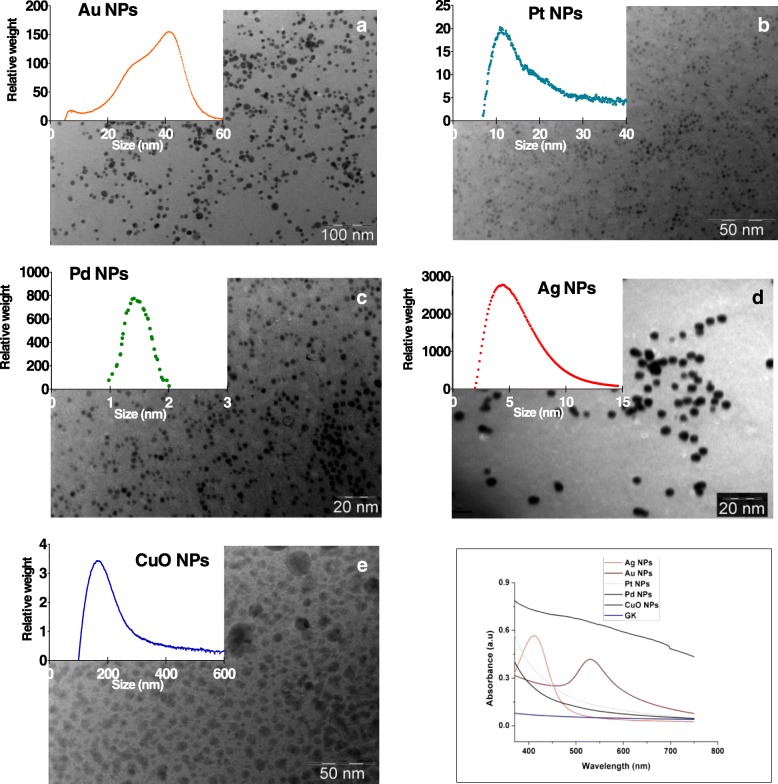
Transmission electron microscopy images of **a** Au, **b** Pt, **c** Ag, **d** Pd and **e** CuO nanoparticles synthesised using gum karaya and their corresponding metal salts. **a**, **b**, **c**, **d** and **e** graph inserts show the peak particle size distribution by nanoparticle weight in algal media, as determined by differential centrifugal sedimentation. (F) UV–Vis spectra for the Au, Ag, Pt, Pd and CuO nanoparticles

TEM images of Ag, Au, Pt, Pd and CuO NPs synthesised using GK show well-separated, spherical NPs with diameters ranging from 2 to 100 nm (Fig. 1a–e). Aqueous colloidal NPs solutions examined under UV–Vis spectroscopy (Fig. 1f) displayed distinct surface plasmon resonance at 412 and 525 nm, consistent with the formation of Au and Ag NPs within the GK network. No distinct surface plasmon resonances were observed for Pt, Pd or CuO NPs. UV–Vis measurements after 6 months confirmed the stability of all NPs, the spectra displaying a single peak with a similar average size as the freshly synthesised NPs (Additional file 1: Figure S2).

### Characterisation of NPs in Algal Exposure Medium

NPs size, based on weight distribution, ranged from 180 to 5 nm as follows: CuO > Au > Pt > Ag > Pd. All NPs were negatively charged at pH 7 (Table 1 and Additional file 1: Figure S3). Pt, Ag and CuO NPs had highest ionic metal concentrations (33–36 μg/L), and Au and Pt NPs the lowest (6–7 μg/L) (Table 1). The ionic forms of metals were detected in algal medium (Table 1).

**Table 1:** Nanoparticle size, zeta-potential and ionic metal concentration determined in TAPx4 medium at a concentration of 20 mg/L. Size and zeta-potential are presented ±standard deviation

NPs	Size (nm)	Zeta (ζ) potential (mV)	Ionic metal (μg/L)
Au	42 ± 0.3	− 24.4 ± 0.8	6
Pt	12 ± 0.3	− 9.1 ± 2.4	36
Ag	5 ± 0.2	− 5.04 ± 2.3	33
Pd	1.5 ± 0.2	− 21.9 ± 2.3	7
CuO	180 ± 0.3	− 28.6 ± 0.9	35

### Effect on Algal Growth

**Fig. 2:**
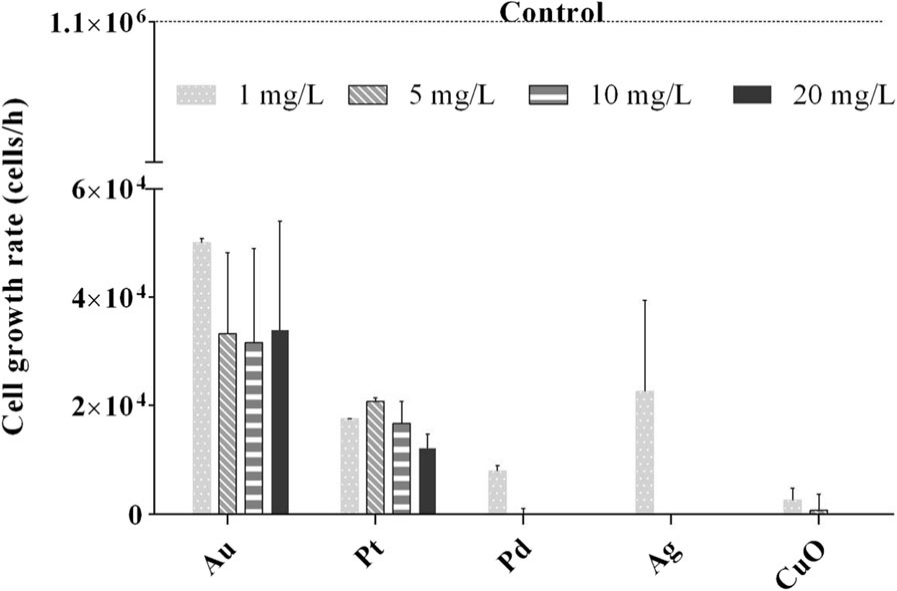
Growth rate of *Chlamydomonas reinhardtii* exposed to Au, Pt, Pd, Ag and CuO metal and metal oxide nanoparticles (1, 5, 10 and 20 mg/L). The growth rate for the non-exposed control (algal culture) was 1 × 10^6^ cells/h after 24 h. The error bars represent the standard deviation of repeated experiments using duplicate samples

Unaffected *C. reinhardtii* culture had a growth rate of 1 × 10^6^ cells/h. In the presence of 1 mg/L of Ag, Pd and CuO NPs, growth rate decreased sharply to 2.2 × 10^4^, 1.7 × 10^4^ and 0.2 × 10^4^ cells/h, respectively (*P* < 0.001). As NP concentration increased further, algal growth was completely inhibited (Fig. 2). When algae were exposed to Au and Pt NPs, growth rate was also significantly reduced compared to the control (*P* < 0.001), but increasing concentrations did not increase the effect.

### Generation of Oxidative Stress in Cells

**Fig. 3:**
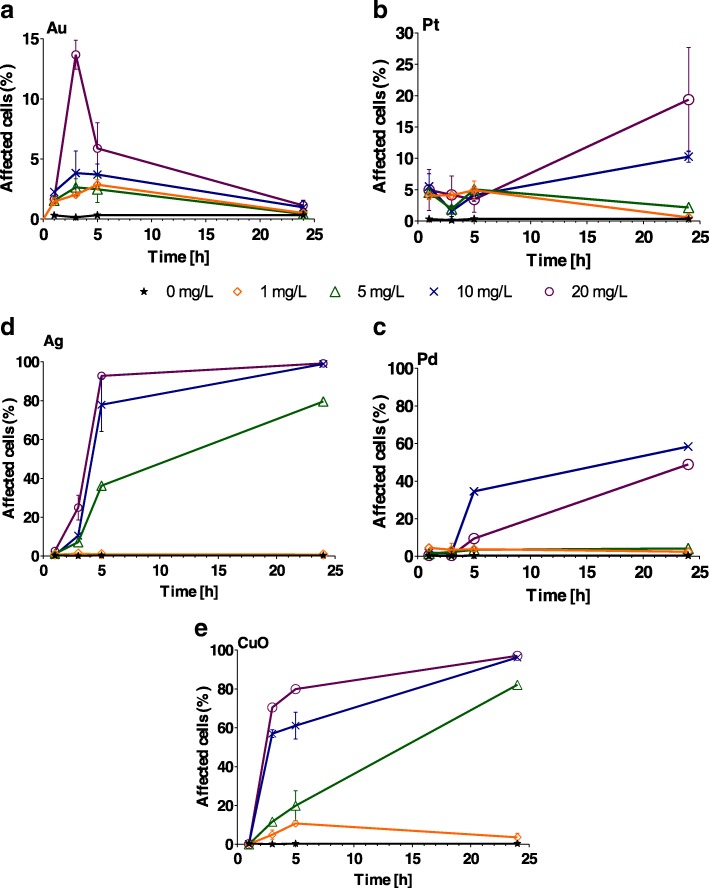
Percentage of *Chlamydomonas reinhardtii* cells exhibiting oxidative stress following exposure to increasing concentrations (1, 5, 10, and 20 mg/L) of **a** Au, **b** Pt, **c** Pd, **d** Ag and **e** CuO nanoparticles after 1, 3, 5 and 24 h. The error bars represent the standard deviation of repeated experiments using duplicate samples. Note different *y*-axis scales for Au and Pt

Oxidative stress varied depending on the NP type (Fig. 3). The highest effect, with almost 100% of cells affected, was caused by 5–20 mg/L of Ag and CuO NPs (Fig. 3d, e and Additional file 1: Table S2). When algal cells were exposed to Au NPs, oxidative stress was much lower, with mostly < 10% of cells affected . The highest concentrations of Au NPs (20 mg/L) affected only 15% of cells (*P* < 0.001). The percentage of stressed cells decreased gradually over time, with no oxidative stress detected after 24 h for all Au concentrations tested (Fig. 3a). Pt NPs caused oxidative stress in less than 8% of algal cells during the first 5 h of exposure (Fig. 3b). Only at concentrations of 10 and 20 mg/L was stress generated in 10 and 19% of cells, respectively, after 24 h (*P* < 0.001; Additional file 1: Table S2), with no stress detected at lower concentrations (*P* > 0.1) after 24 h exposure (Fig. 3b). Exposure to 1 mg/L of Ag NPs failed to induce oxidative stress in algal cells over a 24-h period (*P* > 0.9). However, exposure to 5 mg/L resulted in oxidative stress after 5 h, and exposure to 10 and 20 mg/L resulted in oxidative stress after 3 h. After 24 h exposure to Ag NPs, 100% of cells were stressed (*P* < 0.001; Fig. 3c and Additional file 1: Table S2). CuO NPs induced significant (*P* < 0.001) oxidative stress in algal cells more quickly (3 h) than the other metal NPs tested at 10 and 20 mg/L (Additional file 1: Table S2), except for Ag NPs. Oxidative stress was already significant at 5 mg/L after 5 h. All concentrations (> 5 mg/L) had a significant effect on cell oxidative stress (Fig. 3d, e). As a complementary parameter, we also determined the abiotic ROS generated by the NPs. In contrast to *C. reinhardtii* growth rate and percentage of *C. reinhardtii* cells exhibiting oxidative stress, Au NPs only generated a slight increase in abiotic ROS (*P* > 0.05; Additional file 1: Figure S4).

### Effect on Algal Membrane Integrity

**Fig. 4:**
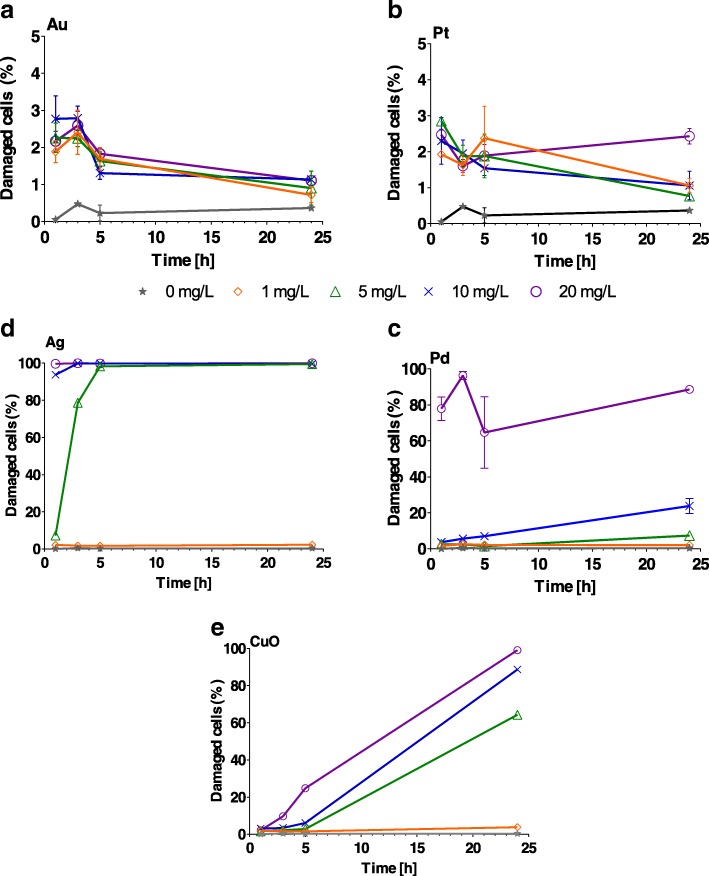
Percentage of *Chlamydomonas reinhardtii* cells with damaged membranes following exposure to increasing concentrations (1, 5, 10 and 20 mg/L) of **a** Au, **b** Pt, **c** Pd, **d** Ag and **e** CuO nanoparticles after 1, 3, 5 and 24 h. The error bars represent the standard deviation of repeated experiments using duplicate samples. Note different *y*-axis scales for Au and Pt

Au and Pt NPs caused significant (*P* < 0.001) cell membrane damage at all concentrations from 1 to 5 h (Additional file 1: Table S3); however, no significant effect (*P* > 0.05) was observed after 24 h (Fig. 4a, b). In the case of Ag NPs, 100% of cells were damaged (*P* < 0.001) after 1 h exposure to 1–20 mg/L (Fig. 4c, Additional file 1: Table S3, Ag). The percentage of cell membranes damaged following exposure to 1 and 5 mg/L Pd NPs (Additional file 1: Table S3, Pd) was comparable with that for the control over 24 h (*P* > 0.4). On the other hand, significant damage (*P* < 0.001) was observed after 24 h exposure to 20 mg/L Pd NPs (Fig. 4d). The effect of CuO increased with increasing concentration and time, achieving its highest impact after 24 h (Fig. 4e and Additional file 1: Table S3).

### Effect on Chlorophyll (*Chl*) Fluorescence

**Fig. 5:**
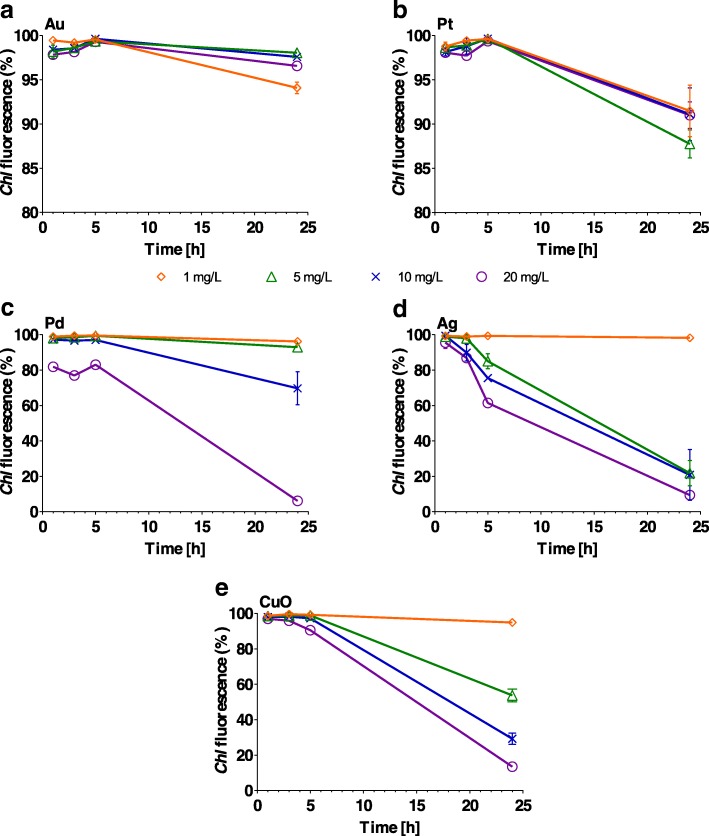
Percentage of *Chlamydomonas reinhardtii* cells with chlorophyll (*Chl*) fluorescence following exposure to increasing concentrations (1, 5, 10 and 20 mg/L) of **a** Au, **b** Pt, **c** Pd, **d** Ag and **e** CuO nanoparticles after 1, 3, 5 and 24 h. The error bars represent the standard deviation of repeated experiments using duplicate samples

*Chl* fluorescence was not significantly affected (*P* > 0.1) by Au NPs at any concentration over the 24-h period and by Pt over the 5-h period (Fig. 5a, b and Additional file 1: Table S4). On the other hand, Ag, Pd and CuO NPs strongly inhibited (*P* < 0.001) *Chl* fluorescence with increasing concentration and exposure time, e.g. *Chl* fluorescence was reduced from 98% (1 h) to 22% (24 h) (*P* < 0.001) when algal cells were grown in the presence of 5 mg/L Ag (Additional file 1: Table S4). A similar reduction in fluorescence was also observed for 10 and 20 mg/L Ag, with levels dropping to 20 and 9% (*P* < 0.001), respectively (Fig. 5c). CuO and Pd NPs (both 20 mg/L) caused a sharp decline in *Chl* fluorescence after 24 h (*P* < 0.001). There was no observable effect (*P* > 0.1), however, for 1 or 5 mg/L of Pd and for 1 mg/L of Ag and CuO NPs (Fig. 5c–e).

### Effect of NPs on Algal Photosystem II

**Fig. 6:**
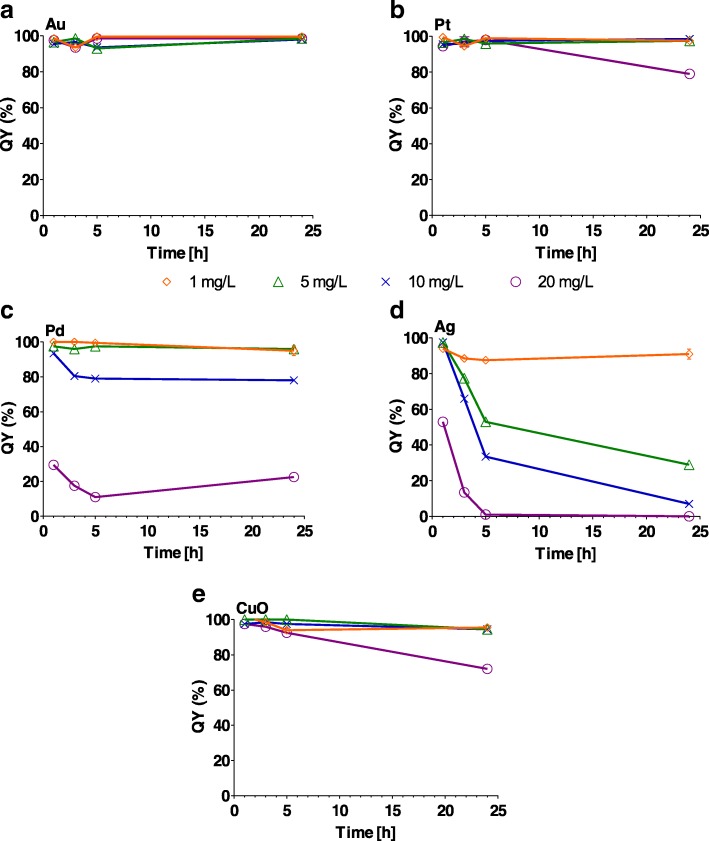
Effect of **a** Au, **b** Pt, **c** Pd, **d** Ag and **e** CuO nanoparticles (1, 5, 10 and 20 mg/L) on photosystem II efficiency (QY %) after 1, 3, 5 and 24 h. One hundred percent on the *y*-axis represents the QY of the control algal culture without nanoparticles. The error bars represent the standard deviation of repeated experiments of duplicated samples

Au, Pt and CuO NPs had a slight significant effect (*P* < 0.05) on photosystem II QY at some time points over the 24-h period at concentrations ranging from 1 to 20 mg/L (Fig. 6 and Additional file 1: Table S5). On the other hand, QY was significantly reduced (*P* < 0.001) after just 1 h following contact with Ag NPs at all concentrations (Fig. 6c and Additional file 1: Table S5). Pd and CuO NPs resulted in a significant reduction in QY at the highest concentration of 20 mg/L (Fig. 6d, e and Additional file 1: Table S5).

## Discussion

In the present work, we aimed to explore elimination of the production of toxic waste during synthesis of metal and metal oxide nanomaterials in implementing the green chemistry approach [16, 57, 58], the major emphasis being on the use of environmentally benign dispersants and renewable and biodegradable materials. We successfully used GK, a natural, renewable and biodegradable material for the synthesis and stabilisation of a range of NPs. Using DI water as a solvent, the functional groups present in GK (i.e. –OH and –COO–) acted as reducing agents and the GK polymer itself acted as a capping agent for the NPs formed, thereby enabling green synthesis of NPs [59, 68]. The NPs synthesised in our study (Au, Pt, Pd, Ag and CuO) were comparable in terms of size, stability and cost-effectiveness to other green synthesised NPs from previous studies [13, 69].

We then used a range of nanoscale concentrations (1–20 mg/L) related to expected or recorded environmental concentrations [39, 71–73] to assess the biological effect of the NPs on *C. reinhardtii* using endpoints such as algal growth, membrane integrity, *Chl* fluorescence photosystem II efficiency and oxidative stress. Our results revealed two distinct groupings: Au and Pt NPs having little or no effect on the alga, and Ag, Pd and CuO NPs displaying a strong effect on almost all endpoints (Additional file 1: Table S6). Toxicity studies of metal or metal oxide NPs have identified several key physico-chemical characteristics of NPs that can be related to their toxicity, including composition, coating, size, shape and homo- or heteroaggregation [69, 74–78]. Moreover, dissolved metal (ionic form) toxicity has previously been demonstrated on algae using a range of criteria, including intracellular ROS generation, *Chl* depletion and photosynthesis inhibition [79–81]. We clearly detected ROS generation and an effect on growth, *Chl* production and photosystem II following exposure to Ag, Pd and CuO NPs.

While Pd NPs have usually been considered a toxic group, they have not been widely studied and they have only recently been recognised as important antibacterial NPs [41]. It is generally believed that the small size (1.5–3 nm) of Pd NPs contributes toward their antibacterial attributes, possibly facilitating transport to cells through bacteria or algal cell wall pores, which have diameters ranging from 5 to 20 nm [82, 83]. In our study, Pd NPs of 1.5 nm mean size could directly enter algal cell walls and cause damage when releasing ions in the cell membrane and chloroplasts (*Chl* fluorescence, PS II, ROS). There is clear evidence that soluble Pd salt was able to enter *P. subcapitata* cells, where Pd precipitates were mostly formed in chloroplasts [78] which could increase generation of ROS and thus oxidative stress. It was also reported that Pd NPs (127 nm *z*-average hydrodynamic size) were less toxic toward *P. subcapitata* than soluble Pd salt [69] maybe due to larger size of NPs that could not directly enter the cells, while Pd salt could. On the other hand, Pd NPs could form hetero-aggregates with algal cells leading to physical entrapment. Surprisingly, the entrapment is not inevitably lethal because the cells could recover their growth after transfer to clean medium [69].

Numerous studies have shown that Ag NPs toxicity to algae was mainly driven by Ag ions dissolved in the exposure medium rather than Ag NPs and also depended on Ag NPs coatings and sizes [80, 84–89]. Our study revealed high toxicity of Ag NPs thus suitable for algicidal applications. The ionic Ag and/or Ag NPs (5 nm) could directly enter algal cells [90], causing damage to the cell membranes and other cellular compartments by ROS formation. Moreover, Ag NPs could damage algal cells by direct interaction between NPs and algal cells [72] or the type of NPs coating could play a significant role. For example, dexpanthenol, polyethylene glycol and polyvinyl polypyrrolidone coatings caused a similar effect as AgNO_3_ on *C. reinhardtii*, while carbonate, chitosan, and citrate decreased the Ag effect on photosynthesis [87]. Our Ag NPs showed strong effect toward *C. reinhardtii* regardless GK coating.

The ecotoxicity of CuO NPs has been extensively studied [36, 47–49, 69, 91]. We observed CuO NPs harming cell membranes right after 1 h, while the ROS elevated after 3 h at concentrations higher than 5 mg/L and also *Chl* fluorescence substantially decreased over 24 h. It is possible that the CuO NPs (or ionic Cu)-damaged membranes could increase further uptake of Cu and oxidative stress in the *C. reinhardtii* cells [91] where observed hetero-aggregation of NPs and the cells (data not shown) could even enhance this interaction. von Moos et al. [36] stated that free Cu^2+^ or the NPs themselves were the main mediators of toxicity toward *C. reinhardtii* , while Cheloni et al. [47] believed ion Cu at lower CuO NPs concentrations was the driving force, being unable to clarify the contribution of dissolved Cu in CuO NPs . This was probably elucidated by other study revealed much stronger effect of soluble ionic Cu and soluble fraction of CuO NPs on *P. subcapitata* than bare CuO NPs [69].

Au NPs slightly increased membrane impairment and oxidative stress after 3 and 5 h, but these effects disappeared after 24 h. Interestingly, abiotic ROS were constantly generated during whole 24 h study contrary to all other NPs. We assume that stable conditions allowed the cells to cope with such small level of stress. Previous study has reported a range of EC50 values for dissolved Au on *C. reinhardtii* of between 5.9 and 1.7 mg/L, depending on exposure time [92]. In our opinion, almost any Au NP toxicity would not have been exacerbated or affected by the degree of ion Au and would have had nearly no bearing on any of the criteria adopted for our experiments. Moreover, Au NPs seemed to be well dispersed in exposure media and we did not observe any aggregates or direct interactions with the *C. reinhardtii* cells (data not shown).

We found that Pt NPs caused slight *Chl* and a growth rate decrease after 24 h for all concentrations. These not so pronounced effects could be caused by both ionic Pt and Pt NPs. Up to now, there has been only limited knowledge about the toxicity of Pt NPs on algae. For example, Pt NPs decreased growth rate, and *Chl* fluorescence and oxidative stress on *P. subcapitata* and *C. reinhardtii* [39, 40]. The latter authors also suggested that the toxicity of Pt NPs might be only partly attributed to dissolved form of Pt in the case of *P. subcapitata* and that also the shading effect might influence toxicity [40]. In our study, we did not find such evidence.

## Conclusions

Green-synthesised metal and metal oxide NPs were produced at nanoscale sizes of 42 nm (Au), 12 nm (Pt), 1.5 nm (Pd), 5 nm (Ag) and 180 nm (CuO): all with a negative charge. GK, a natural hydrocolloid, was successfully applied as a safe, cost-effective stabiliser and showed no aggregation (all NPs) after 6 months at + 4 °C. The biological effect (algal growth, membrane integrity, oxidative stress, *Chl* fluorescence and photosystem II efficiency) of these NPs was investigated on green alga *C. reinhardtii*. All NPs had a significant effect on algal growth rate; however, Au and Pt NPs inhibited algal growth far less than the other NPs (Pd, Ag and CuO). In terms of other biological effects, Pd, Ag and CuO NPs caused significant cell membrane damage, highly affected *Chl* fluorescence and caused oxidative stress. Ag and Pd NPs mostly inhibited photosystem II, while it was not much affected by CuO (only the highest concentration of 20 mg/L significantly decreased QY) and Au or Pt. Generally, metal and metal oxide NPs were successfully synthesised following green chemistry rules, without harmful side-products and showing high stability. Some could find reasonable application in algicides (Ag and CuO) or antimicrobial surfaces (Pd, Ag and CuO), while Au and Pt proved to be almost non-toxic to green alga *C. reinhardtii*.

## Additional file

Additional file 1: **Table S1.** Ingredients of algal growth medium (TAPx4). **Table S2.** *P* values of affected cells (generation of oxidative stress). **Table S3.** P values of damaged cell membrane. **Table S4.** P values of effected cells via *Chl* fluorescence. **Table S5.** P values of effected cells via efficiency of photosystem II. **Table S6.** Biological effects of NPs on algal cells in the orders. **Figure S1.** An example analysis of flow cytometry data: Side scatter vs Forward scatter: (a) Algae stock, (b) NPs; Count vs. auto-fluorescence (FL3/*Chla*): (c) Algae stock, (d)NPs; Green ROS marker with heated algae + NMs: (e) Count vs. FL1/ROS, (f) FL1/ROS vs. FL3/*Chla*; Propidium iodide (PI) marker with heated algae + NMs: (g) Count vs. FL2/PI, (h) FL2/PI vs. FL3/*Chla*. **Figure S2.** Stability of Au, Ag, Pt, Pd and CuO; stability after 6 months was recorded by UV–Vis spectroscopy. **Figure S3.** Zeta-potential of NPs after 1 h and 24 h presence in algal growth TAPx4 medium. **Figure S4.** Abiotic generation of ROS by NPs 1, 3, 5 and 24 h exposure to the different concentrations. Positive control was obtained by incubating the probe with a mixture of 1 mM FeSO_4_ and 0.5% H_2_O_2_. The H_2_DCF probe in the exposure medium incubated in the dark was used as a blank control. Data are shown as a ratio between the fluorescent values obtained for the samples and those obtained for the blank control. Error bars represent the standard deviations of triplicate measurements. Dotted lines indicate the background. (DOCX 468 kb)
