# Supplementary material for: Green Synthesis of Metal and Metal Oxide Nanoparticles and Their Effect on the Unicellular Alga Chlamydomonas reinhardtii
Source: Nanoscale Res Lett. 2018 May 23;13:159. doi: 10.1186/s11671-018-2575-5 (PMC5966349; doi:10.1186/s11671-018-2575-5)
Supplement: Supplementary file 1 — Table S1. Ingredients of algal growth medium (TAPx4). Table S2. P values of affected cells (generation of oxidative stress). Table S3. P values of damaged cell membrane. Table S4. P values of effected cells via Chl fluorescence. Table S5. P values of effected cells via efficiency of photosystem II. Table S6. Biological effects of NPs on algal cells in the orders. Figure S1. An example analysis of flow cytometry data: Side scatter vs Forward scatter: (a) Algae stock, (b) NPs; Count vs. auto-fluorescence (FL3/Chla): (c) Algae stock, (d)NPs; Green ROS marker with heated algae + NMs: (e) Count vs. FL1/ROS, (f) FL1/ROS vs. FL3/Chla; Propidium iodide (PI) marker with heated algae + NMs: (g) Count vs. FL2/PI, (h) FL2/PI vs. FL3/Chla. Figure S2. Stability of Au, Ag, Pt, Pd and CuO; stability after 6 months was recorded by UV–Vis spectroscopy. Figure S3. Zeta-potential of NPs after 1 h and 24 h presence in algal growth TAPx4 medium. Figure S4. Abiotic generation of ROS by NPs 1, 3, 5 and 24 h exposure to the different concentrations. Positive control was obtained by incubating the probe with a mixture of 1 mM FeSO4 and 0.5% H2O2. The H2DCF probe in the exposure medium incubated in the dark was used as a blank control. Data are shown as a ratio between the fluorescent values obtained for the samples and those obtained for the blank control. Error bars represent the standard deviations of triplicate measurements. Dotted lines indicate the background. (DOCX 468 kb) [file 11671_2018_2575_MOESM1_ESM.docx]

#### Additional file

**Green synthesis of metal and metal oxide nanoparticles and their effects on the unicellular alga *Chlamydomonas reinhardtii***

**Nhung H. A. Nguyen^1^, Vinod Vellora Thekkae Padil**^1^, **Vera I. Slaveykova^2^, Miroslav Černík^1^, Alena Ševců^1^**

^1^Technical University of Liberec, Institute for Nanomaterials, Advanced Technologies and Innovation, and Faculty of Mechatronics, Studentska 2, Liberec, Czech Republic.

^2^University of Geneva, Faculty of Sciences, Earth and Environmental Sciences, Institute F.-A. Forel, Uni Carl Vogt, 66 Bvd Carl-Vogt, CH-1211 Geneva, Switzerland.

**CONTENTS**

**1. MATERIALS AND METHODS**

1.1 Table S1. Ingredients of algal growth medium TAPx4.

**2. RESULTS**

2.1 Figure S1. Analysis of flow cytometry data: Side scatter vs Forward scatter: (a) Algae stock, (b) NPs; Count vs. Chlorophyll fluorescence (FL3/*Chl*): (c) Algae stock, (d) NPs; Green ROS marker with heated algae + NPs: (e) Count vs. FL1/ROS, (f) FL1/ROS vs. FL3/*Chl*; Propidium iodide (PI) marker with heated algae + NPs: (g) Count vs. FL2/PI, (H) FL2/PI vs. FL3/*Chl*.

2.2 Figure S2. Stability of Au, Ag, Pt, Pd and CuO, stability after 6 months was recorded by UV–vis spectra.

2.3 Figure S3. Zeta-potential of NPs after 1h and 24h presence in algal growth TAPx4 medium.

2.5 Figure S4. Abiotic generation of ROS by NPs after 1, 3, 5 and 24h exposure to the different concentrations. Positive control was obtained by incubating the probe with a mixture of 1 mM FeSO4 and 0.5% H_2_O_2_. The H_2_DCF probe in the exposure medium, incubated in the dark, was used as a blank control.

2.5 P values of affected cells (Table S2), damaged cells (Table S3), *Chl* fluorescence (Table S4), efficency of photosystem II (Table S5) and Biological effects of NPs on algal cells in the orders (Table S6).

**Table S1.** Ingredients of algal growth medium (TAPx4)

| **Components** | **Concentration (mol/L)** |
| --- | --- |
| **Phosphate** | |
| KH_2_PO_4_ | 1.01E-04 |
| K_2_HPO_4_ | 1.49E-04 |
| **Ammonium** | |
| NH_4_NO_3_ | 1.68E-03 |
| CaCl_2_, 2 H_2_O | 8.50E-05 |
| MgSO_4_, 7 H_2_O | 1.02E-04 |
| **Metals** | |
| H_3_BO_3_ | 4.75E-05 |
| ZnSO_4_, 7 H_2_O | 1.90E-05 |
| MnCl_2_, 4 H_2_O | 6.25E-05 |
| FeSO_4_, 7 H_2_O | 4.50E-06 |
| CoCl_2_, 6 H_2_O | 1.67E-06 |
| CuSO_4_, 5 H_2_O | 1.57E-06 |
| (NH4)_6_Mo_7_O_24_,4 H_2_O | 2.23E-06 |
| Na_2_EDTA, 2 H_2_O | 3.36E-05 |
| **TRIS acetate** | |
| CH_3_COOH | 4.30E-03 |
| TRIS | 5.00E-03 |

| Side scatter vs Forward scatter | 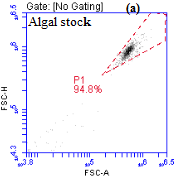 | 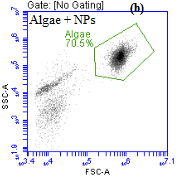 |
| --- | --- | --- |
| Auto-fluorescence (FL3/*Chla*) | 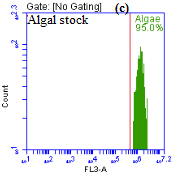 | 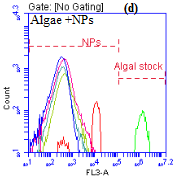 |
| CellRox Green (ROS) marker (FL1/ROS) | 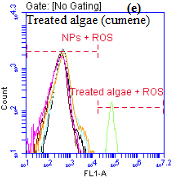 | 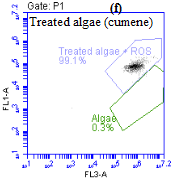 |
| Propidium iodide (PI) marker (FL2/PI) | 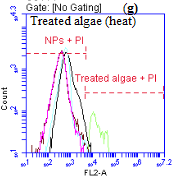 | 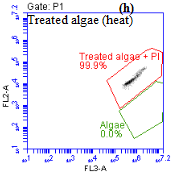 |

**Figure S1.** An example analysis of flow cytometry data: Side scatter vs Forward scatter: (a) Algae stock, (b) NPs; Count vs. auto-fluorescence (FL3/*Chla*): (c) Algae stock, (d)NPs; Green ROS marker with heated algae + NMs: (e) Count vs. FL1/ROS, (f) FL1/ROS vs. FL3/*Chla*; Propidium iodide (PI) marker with heated algae + NMs: (g) Count vs. FL2/PI, (h) FL2/PI vs. FL3/*Chla*.


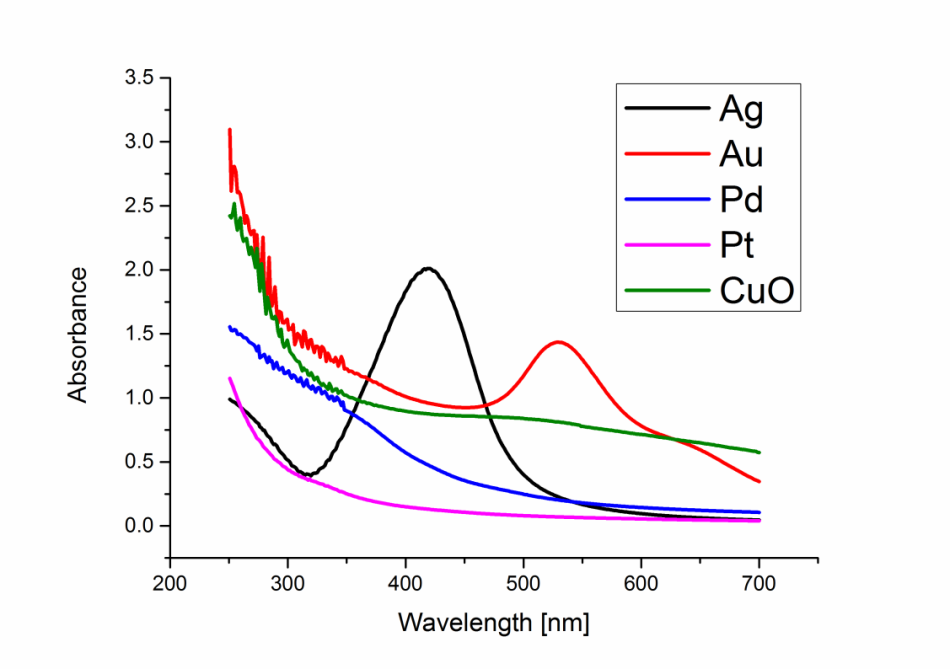


**Figure S2:** Stability of Au, Ag, Pt, Pd and CuO; stability after 6 months was recorded by

UV–vis spectroscopy.

**Figure S3.** Zeta-potential of NPs after 1h and 24h presence in algal growth TAPx4 medium.

**Figure S4.** Abiotic generation of ROS by NPs 1, 3, 5 and 24h exposure to the different concentrations. Positive control was obtained by incubating the probe with a mixture of 1 mM FeSO_4_ and 0.5% H_2_O_2_. The H_2_DCF probe in the exposure medium incubated in the dark was used as a blank control. Data are shown as a ratio between the fluorescent values obtained for the samples and those obtained for the blank control. Error bars represent the standard deviations of triplicate measurements. Dotted lines indicate the background.

**Table S2. P values of affected cells (generation of oxidative stress)**

Pt < Au < Ag ≤ Pd < CuO

| Au | Time 1h | Time 3h | Time 5h | Time 24h |
| --- | --- | --- | --- | --- |
| 0 mg/L vs. 1 mg/L | 0.4308 | 0.0894 | 0.0168 | 0.9980 |
| 0 mg/L vs. 5 mg/L | 0.3771 | 0.0179 | 0.0453 | 0.9998 |
| 0 mg/L vs. 10 mg/L | 0.0781 | 0.0006 | 0.0015 | 0.8272 |
| 0 mg/L vs. 20 mg/L | 0.3094 | < 0.0001 | < 0.0001 | 0.7093 |

| Pt | Time 1h | Time 3h | Time 5h | Time 24h |
| --- | --- | --- | --- | --- |
| 0 mg/L vs. 1 mg/L | 0.32 | 0.26 | 0.17 | 0.99 |
| 0 mg/L vs. 5 mg/L | 0.21 | 0.85 | 0.15 | 0.83 |
| 0 mg/L vs. 10 mg/L | 0.10 | 0.92 | 0.30 | 0.001 |
| 0 mg/L vs. 20 mg/L | 0.16 | 0.23 | 0.47 | < 0.001 |

| Ag | Time 1h | Time 3h | Time 5h | Time 24h |
| --- | --- | --- | --- | --- |
| 0 mg/L vs. 1 mg/L | 0.99 | 0.99 | 0.99 | 0.99 |
| 0 mg/L vs. 5 mg/L | 0.99 | 0.16 | < 0.001 | < 0.001 |
| 0 mg/L vs. 10 mg/L | 0.99 | 0.02 | < 0.001 | < 0.001 |
| 0 mg/L vs. 20 mg/L | 0.91 | < 0.001 | < 0.001 | < 0.001 |

| Pd | Time 1h | Time 3h | Time 5h | Time 24h |
| --- | --- | --- | --- | --- |
| 0 mg/L vs. 1 mg/L | 0.006 | 0.036 | 0.02 | 0.25 |
| 0 mg/L vs. 5 mg/L | 0.74 | 0.27 | 0.05 | 0.013 |
| 0 mg/L vs. 10 mg/L | 0.52 | 0.51 | < 0.001 | < 0.001 |
| 0 mg/L vs. 20 mg/L | 0.99 | 0.99 | < 0.001 | < 0.01 |

| CuO | Time 1h | Time 3h | Time 5h | Time 24h |
| --- | --- | --- | --- | --- |
| 0 mg/L vs. 1 mg/L | 0.99 | 0.21 | 0.019 | 0.52 |
| 0 mg/L vs. 5 mg/L | 0.99 | 0.008 | < 0.001 | < 0.001 |
| 0 mg/L vs. 10 mg/L | > 0.99 | < 0.001 | < 0.001 | < 0.001 |
| 0 mg/L vs. 20 mg/L | > 0.99 | < 0.001 | < 0.001 | < 0.001 |
|  |  |  |  |  |
|  |  |  |  |  |

**Table S3. P values of damaged cell membrane**

Pd < Au ≤ Pt < CuO < Ag

| Au | Time 1h | Time 3h | Time 5h | Time 24h |
| --- | --- | --- | --- | --- |
| 0 mg/L vs. 1 mg/L | < 0.001 | < 0.001 | 0.004 | 0.60 |
| 0 mg/L vs. 5 mg/L | < 0.001 | < 0.001 | 0.006 | 0.25 |
| 0 mg/L vs. 10 mg/L | < 0.001 | < 0.001 | 0.071 | 0.06 |
| 0 mg/L vs. 20 mg/L | < 0.001 | < 0.001 | 0.002 | 0.078 |

| Pt | Time 1h | Time 3h | Time 5h | Time 24h |
| --- | --- | --- | --- | --- |
| 0 mg/L vs. 1 mg/L | 0.001 | 0.012 | < 0.001 | 0.17 |
| 0 mg/L vs. 5 mg/L | < 0.001 | 0.024 | 0.005 | 0.60 |
| 0 mg/L vs. 10 mg/L | < 0.001 | 0.014 | 0.042 | 0.17 |
| 0 mg/L vs. 20 mg/L | < 0.001 | 0.014 | 0.004 | < 0.001 |

| Ag | Time 1h | Time 3h | Time 5h | Time 24h |
| --- | --- | --- | --- | --- |
| 0 mg/L vs. 1 mg/L | 0.003 | 0.02 | 0.01 | 0.008 |
| 0 mg/L vs. 5 mg/L | < 0.001 | < 0.001 | < 0.001 | < 0.001 |
| 0 mg/L vs. 10 mg/L | < 0.001 | < 0.001 | < 0.001 | < 0.001 |
| 0 mg/L vs. 20 mg/L | < 0.001 | < 0.001 | < 0.001 | < 0.001 |

| Pd | Time 1h | Time 3h | Time 5h | Time 24h |
| --- | --- | --- | --- | --- |
| 0 mg/L vs. 1 mg/L | 0.99 | 0.97 | 0.98 | 0.99 |
| 0 mg/L vs. 5 mg/L | 0.95 | 0.97 | 0.99 | 0.41 |
| 0 mg/L vs. 10 mg/L | 0.86 | 0.66 | 0.44 | 0.004 |
| 0 mg/L vs. 20 mg/L | < 0.001 | < 0.001 | < 0.001 | < 0.001 |

| CuO | Time 1h | Time 3h | Time 5h | Time 24h |
| --- | --- | --- | --- | --- |
| 0 mg/L vs. 1 mg/L | 0.067 | 0.102 | 0.047 | < 0.001 |
| 0 mg/L vs. 5 mg/L | 0.017 | 0.011 | 0.002 | < 0.001 |
| 0 mg/L vs. 10 mg/L | < 0.001 | < 0.001 | < 0.001 | < 0.001 |
| 0 mg/L vs. 20 mg/L | 0.021 | < 0.001 | < 0.001 | < 0.001 |

**Table S4. P values of effected cells via *Chl* fluorescence**

Au < Pt ≤ Pd < CuO < Ag

| Au | Time 1h | Time 3h | Time 5h | Time 24h |
| --- | --- | --- | --- | --- |
| 0 mg/L vs. 1 mg/L | 0.53 | 0.84 | 0.32 | < 0.001 |
| 0 mg/L vs. 5 mg/L | 0.09 | 0.80 | 0.70 | 0.94 |
| 0 mg/L vs. 10 mg/L | 0.26 | 0.70 | 0.22 | 0.84 |
| 0 mg/L vs. 20 mg/L | 0.01 | 0.12 | 0.82 | 0.05 |

| Pt | Time 1h | Time 3h | Time 5h | Time 24h |
| --- | --- | --- | --- | --- |
| 0 mg/L vs. 1 mg/L | 0.99 | 0.97 | 0.94 | < 0.001 |
| 0 mg/L vs. 5 mg/L | 0.99 | 0.99 | 0.93 | < 0.001 |
| 0 mg/L vs. 10 mg/L | 0.84 | 0.99 | 0.93 | < 0.001 |
| 0 mg/L vs. 20 mg/L | 0.81 | 0.66 | 0.99 | < 0.001 |

| Ag | Time 1h | Time 3h | Time 5h | Time 24h |
| --- | --- | --- | --- | --- |
| 0 mg/L vs. 1 mg/L | 0.99 | 0.99 | 0.9999 | 0.99 |
| 0 mg/L vs. 5 mg/L | 0.99 | 0.99 | 0.0079 | < 0.001 |
| 0 mg/L vs. 10 mg/L | 0.99 | 0.10 | < 0.001 | < 0.001 |
| 0 mg/L vs. 20 mg/L | 0.79 | 0.02 | < 0.001 | < 0.001 |

| Pd | | Time 1h | Time 3h | Time 5h | Time 24h |
| --- | --- | --- | --- | --- | --- |
| 0 mg/L vs. 1 mg/L | | 0.99 | 0.99 | 0.99 | 0.85 |
| 0 mg/L vs. 5 mg/L | | 0.97 | 0.99 | 0.99 | 0.11 |
| 0 mg/L vs. 10 mg/L | | 0.81 | 0.68 | 0.76 | < 0.001 |
| 0 mg/L vs. 20 mg/L | | < 0.001 | < 0.001 | < 0.001 | < 0.001 |
|  |  |  |  |  |  |
|  |  |  |  |  |  |
|  |  |  |  |  |  |
| CuO | | Time 1h | Time 3h | Time 5h | Time 24h |
| 0 mg/L vs. 1 mg/L | | 0.99 | 0.95 | 0.99 | 0.11 |
| 0 mg/L vs. 5 mg/L | | 0.98 | 0.99 | 0.99 | < 0.001 |
| 0 mg/L vs. 10 mg/L | | 0.83 | 0.93 | 0.51 | < 0.001 |
| 0 mg/L vs. 20 mg/L | | 0.37 | 0.11 | < 0.001 | < 0.001 |

**Table S5. P values of effected cells via efficency of photosystem II**

Au ≤ Pt ≤ CuO < Pd < Ag

| Au | Time 1h | Time 3h | Time 5h | Time 24h |
| --- | --- | --- | --- | --- |
| 0 mg/L vs. 1 mg/L | 0.45 | < 0.001 | 0.97 | 0.97 |
| 0 mg/L vs. 5 mg/L | 0.01 | 0.45 | < 0.001 | 0.45 |
| 0 mg/L vs. 10 mg/L | 0.001 | 0.013 | < 0.001 | 0.22 |
| 0 mg/L vs. 20 mg/L | 0.09 | < 0.001 | 0.45 | 0.45 |

| Pt | Time 1h | Time 3h | Time 5h | Time 24h |
| --- | --- | --- | --- | --- |
| 0 mg/L vs. 1 mg/L | 0.98 | 0.002 | 0.86 | 0.21 |
| 0 mg/L vs. 5 mg/L | 0.05 | 0.62 | 0.02 | 0.21 |
| 0 mg/L vs. 10 mg/L | 0.009 | 0.02 | 0.21 | 0.62 |
| 0 mg/L vs. 20 mg/L | 0.002 | 0.11 | 0.38 | < 0.001 |

| Ag | Time 1h | Time 3h | Time 5h | Time 24h |
| --- | --- | --- | --- | --- |
| 0 mg/L vs. 1 mg/L | 0.002 | < 0.001 | < 0.001 | < 0.001 |
| 0 mg/L vs. 5 mg/L | 0.14 | < 0.001 | < 0.001 | < 0.001 |
| 0 mg/L vs. 10 mg/L | 0.14 | < 0.001 | < 0.001 | < 0.001 |
| 0 mg/L vs. 20 mg/L | < 0.001 | < 0.001 | < 0.001 | < 0.001 |

| Pd | | Time 1h | Time 3h | Time 5h | Time 24h |
| --- | --- | --- | --- | --- | --- |
| 0 mg/L vs. 1 mg/L | | 0.99 | 0.99 | 0.98 | 0.02 |
| 0 mg/L vs. 5 mg/L | | 0.16 | 0.01 | 0.16 | 0.01 |
| 0 mg/L vs. 10 mg/L | | 0.001 | < 0.001 | < 0.001 | < 0.001 |
| 0 mg/L vs. 20 mg/L | | < 0.001 | < 0.001 | < 0.001 | < 0.001 |
|  |  |  |  |  |  |
|  |  |  |  |  |  |
|  |  |  |  |  |  |
| CuO | | Time 1h | Time 3h | Time 5h | Time 24h |
| 0 mg/L vs. 1 mg/L | | 0.27 | 0.27 | < 0.001 | 0.002 |
| 0 mg/L vs. 5 mg/L | | 0.99 | 0.99 | 0.99 | < 0.001 |
| 0 mg/L vs. 10 mg/L | | 0.03 | 0.27 | 0.03 | < 0.001 |
| 0 mg/L vs. 20 mg/L | | 0.03 | 0.006 | < 0.001 | < 0.001 |

**Table S6. Biological effects of NPs on algal cells in the orders.**

| **Table S2. P values of affected cells (generation of oxidative stress)** | Pt < Au < Ag ≤ Pd < CuO |
| --- | --- |
| **Table S3. P values of damaged cell membrane** | Pd < Au ≤ Pt < CuO < Ag |
| **Table S4. P values of effected cells via *Chl* fluorescence** | Au < Pt ≤ Pd < CuO < Ag |
| **Table S5. P values of effected cells via efficency of photosystem II** | Au ≤ Pt ≤ CuO < Pd < Ag |
| **Conclusion** | Au ≤ Pt < Pd < CuO < Ag |
